# Protection from Metabolic Dysregulation, Obesity, and Atherosclerosis by Citrus Flavonoids: Activation of Hepatic PGC1**α**-Mediated Fatty Acid Oxidation

**DOI:** 10.1155/2012/857142

**Published:** 2012-05-30

**Authors:** Erin E. Mulvihill, Murray W. Huff

**Affiliations:** ^1^Vascular Biology Group, Robarts Research Institute, 100 Perth Drive, London, ON, Canada N6A 5K8; ^2^Departments of Biochemistry and Medicine, The University of Western Ontario, London, ON, Canada N6A 5C1

## Abstract

Studies in a multitude of models including cell culture, animal and clinical studies demonstrate that citrus-derived flavonoids have therapeutic potential to attenuate dyslipidemia, correct hyperinsulinemia and hyperglycemia, and reduce atherosclerosis. Emerging evidence suggests the metabolic regulators, PPAR*α* and PGC1*α*, are targets of the citrus flavonoids, and their activation may be at least partially responsible for mediating their metabolic effects. Molecular studies will add significantly to the concept of these flavonoids as viable and promising therapeutic agents to treat the dysregulation of lipid homeostasis, metabolic disease, and its cardiovascular complications.

## 1. Introduction

Metabolic syndrome is a clustering of risk factors including dyslipidemia, hypertension, insulin resistance, and visceral obesity for the development of type 2 diabetes mellitus and premature atherosclerosis [1–3]. While pharmacological agents have been successfully used to target major risk factors including hypertension, plasma cholesterol, and blood sugar, data collected from 1994–2005 suggest that the prevalence of hypertension, diabetes and obesity and the number of people diagnosed with CVD are increasing in all groups of Canadians [4]. The increased risk was most dramatic in the youngest group studied (aged 12–34 years old). In light of the increasing prevalence of metabolic dysregulation, new therapies are required. In the search for new targets and interventions to improve underlying risk factors and prevent the complications of atherosclerosis, attention has turned to nontraditional therapies including a group of naturally-occurring compounds known as flavonoids.

## 2. Flavonoids and Metabolic Disease

Flavonoids are abundant in the human diet as several thousands have been identified in a large variety of beverages and foods [5]. They have been classified based on their structural variation and degree of oxidation [5]. Flavonoid intake is inversely associated with coronary heart disease as men in the highest tertile of flavonoid intake experience the lowest relative risk of mortality from coronary artery disease [6]. An inverse relationship between flavonoid consumption and many risk factors for heart disease [7] including lower blood pressure and improved flow mediated dilatation [8], improved weight management [9], and improved dyslipidemia [8, 10] has also been established, suggesting that flavonoids have multiple targets. In human studies, wide variability has been observed in the effects of flavonoid-rich foods on these markers of cardiovascular disease risk [8]. Understanding the specific targets responsible for risk reduction is difficult to delineate in such a complex system. Therefore, recent attention has turned to purified flavonoids and the examination of their pharmacological properties.

Many studies have estimated flavonoid intake, and while the family of flavan-3-ols including catechins found in tea and chocolate often make up the majority [11], the flavanones also contribute significantly to total daily intake [12]. The citrus flavanones hesperitin and naringenin are members of this family (reviewed in [13]). Naringin, the glycoside form of naringenin found in citrus fruits, is hydrolyzed by intestinal microflora to the flavanone naringenin. Making up a smaller percentage of total intake is the class of flavones which contains the polymethoxylated polyphenols nobiletin and tangeretin, two flavonoids found abundantly in oranges and tangerines [12]. Administration of grapefruit or orange juice to hypercholesterolemic casein-fed rabbits reduced low density lipoprotein cholesterol (LDL-C) and hepatic lipid accumulation suggesting components of citrus may have lipid lowering properties [10]. In rats with streptozotocin- (STZ-) induced diabetes, *i.p.* injection of naringenin decreased blood glucose and improved dyslipidemia [14]. These early data suggested that citrus-derived flavonoids may improve metabolic health in patients with insulin resistance and prevent diabetes and CVD.

While a direct relationship has not been clearly established, a common thread linking many of the features of the metabolic syndrome includes the aberrant tissue deposition of lipid including cholesterol, ceramides, and triglycerides [15]. The dyslipidemia of metabolic syndrome, which is characterized by an overproduction of hepatic very low density lipoproteins (VLDL-) and low high density lipoproteins (HDL-) cholesterol [16], is thought to be a significant contributor to enhanced tissue lipid deposition. Triglyceride (TG) formation is the primary method of metabolic fuel storage for most species [17] and the formation of TG can protect cells from fatty acid (FA) overload and subsequent lipotoxicity [15]. Ectopic lipid accumulation has been described in many tissues including the heart, arteries, liver, muscle, adipose tissue, and pancreas and has been linked to the dysfunction of these tissues in models of insulin resistance and metabolic syndrome [15, 18]. Therefore, one mechanism by which flavonoids have been described to prevent metabolic dysregulation is to limit ectopic lipid accumulation and stimulate fatty acid and glucose utilization.

## 3. PPAR*α*-Mediated Regulation of Metabolism

**Figure 1:**
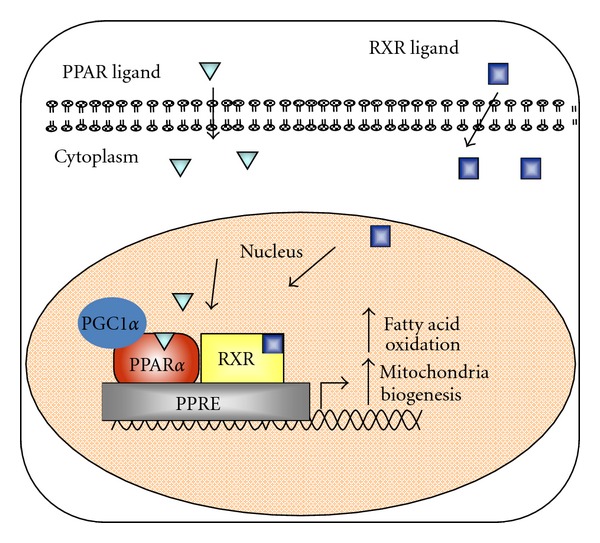
Regulation of gene expression by peroxisome proliferator-activated receptors. The nuclear hormone receptor PPAR*α* induces transcription through formation of a heterodimer with the retinoic X receptor and binding to peroxisome proliferator response elements (most are direct repeats with one intervening nucleotide) in the promoter of genes involved in fatty acid oxidation. PGC1*α* is an important PPAR*α* coactivator in tissues that undergo extensive oxidative metabolism and induce mitochondrial expansion.

Peroxisome proliferator-activated receptor alpha (PPAR*α*) is a member of the family of nuclear hormone receptors which function as ligand activated transcription factors, with a signature type II zinc finger DNA binding motif, to control the expression of specific genes involved in fatty acid utilization [19, 20] (Figure 1). Liver, muscle, kidney, and brown adipose tissue express high levels of PPAR*α* [20, 21]. The endogenous ligands for PPAR*α* activation include fatty acids and their metabolites [22]. PPARs control expression of genes by partnering with RXR and binding to peroxisome proliferator response elements (PPREs) in the promoter of target genes, ultimately resulting in the stimulation of FA oxidation. In mice, receptor agonists cause proliferation of peroxisomes which serve to oxidize long chain fatty acids and detoxify xenobiotic compounds [23]. PPAR*α* is an activator of genes involved in *β*-oxidation including carnitine palmitoyl transferase 1 (*CPT1*α*) *and acyl-CoA oxidase* (ACOX)*. In the liver, fatty acid oxidation is primarily regulated by the level of CPT1 [24]. *CPT1*α** expression is controlled by a complex of transcription factors including PPAR*γ*-coactivator 1 *α* (PGC1*α*) and PPAR*α* [24]. CPT1 is found on the outer surface of the mitochondria and transports fatty acids into the mitochondria by the formation of an acyl-carnitine molecule. Upon entry into the inner side of the mitochondrial membrane, CPT2 removes the carnitine, reforming acyl-CoA [24].

Dynamic regulation of fatty acid oxidation is provided by interaction between acetyl-CoA carboxylase (ACC), a member of the fatty acid synthesis pathway, and CPT1, through the intermediate malonyl-CoA. When malonyl-CoA levels are high, they allosterically inhibit CPT1 and *de novo* lipogenesis continues. When the cell experiences a drop in malonyl-CoA, ACC is inhibited and fatty acid oxidation is stimulated [25, 26]. In liver-specific *Fas^−/−^* mice significant increases in malonyl-CoA were observed to inhibit CPT1*α* resulting in hepatic steatosis [27]. Recently, adenovirus-mediated hepatic expression of a permanently active mutant form of *Cpt1*α** in high-fat fed obese mice, resulted in increased hepatic FA oxidation, thereby reducing hepatic TG content and ameliorating insulin resistance [28]. These studies highlight the importance of CPT1*α*-regulated fatty acid oxidation in maintaining lipid balance in the cell.

Many nuclear receptor cofactors exist which provide additional control of gene expression [29]. In the liver, coactivators important for induction of PPAR*α*-mediated gene transcription and regulation of lipid metabolism include PGC1*α*. PGC1*α* regulates a number of hepatic metabolic pathways, including gluconeogenesis, mitochondrial expansion, and FA oxidation [30]. Once bound to a transcription factor, PGC1*α* allows the interaction of histone modifiers, the transcriptional initiation complex and DNA [31] (Figure 1). Similar to PPAR*α*, hepatic PGC1*α* is induced by fasting, through a cAMP response element [32]. Mice with hepatic-specific knockout of *Pgc1*α** accumulate liver TG, due to impaired *β*-oxidation, and enhanced SREBP1c-stimulated FA and TG synthesis [33, 34]. It is known that PGC1*α* increases genes involved in mitochondrial expansion through induction of the nuclear respiratory factors [31]. Furthermore, PGC1*α* interacts with and coactivates PPAR*α* to transcriptionally regulate the genes involved in FA oxidation and increase palmitate utilization rates [29].

PPAR*α* is most active in response to a drop in blood glucose, whether by fasting or by exercise [20]. In addition to replenishing ATP, the process of FA oxidation also provides the reducing cofactors required for gluconeogenesis and can induce the utilization of FA for the production of ketone bodies [35]. Mice with whole body deficiency of PPAR*α* have decreased capacity for the oxidation FA including palmitic acid [36] and elevated total- and HDL-cholesterol levels [23], after a 48 h fast. Furthermore, *Ppar*α*^−/−^* mice exhibit hypoglycemia, significant lipid accumulation in the liver, increased circulating nonesterified fatty acids, and decreased ketogenesis [37].

Pharmacological activators of PPAR*α*, including the fibrate family of drugs, have demonstrated efficacy for reducing plasma TG and raising HDL in dyslipidemic patients and are often used in patients with insulin resistance. A recent meta-analysis of trials involving hypertriglyceridemic patients demonstrated that fibrates reduce vascular events [38], suggesting a renewed interest in the activation of PPAR*α* to prevent CVD in select patients with dyslipidemia.

## 4. Regulation of Lipid Metabolism and Correction of Dyslipidemia by Citrus Flavonoids

Like fibrates, many citrus-derived flavonoids exhibit lipid-lowering properties. The consumption of 1-2 grapefruits/day significantly reduced cholesterol and triglyceride in hypercholesterolemic patients suffering from coronary artery disease [39, 40]. In a clinical trial involving hypercholesterolemic patients, the citrus flavonoid naringin (400 mg/day) was shown to reduce LDL-cholesterol by 17% and triglycerides by 14% [41]. However, in other recent human trials, capsules of hesperitin (800 mg/day) or naringin (500 mg/day) produced no lipid lowering benefit in moderately hypercholesterolemic individuals [42].

In more mechanistic studies, it was demonstrated in cultured hepatoma cells that naringenin reduces CE availability for lipoprotein assembly and secretion through inhibition of ACAT and MTP activities [43, 44]. However, subsequent studies established that the reduction in CE accumulation within the lumen of the endoplasmic reticulum (ER) did not mediate the naringenin-induced inhibition of apolipoprotein B100 (apoB100) assembly. Instead it was the reduction in TG accumulation within the ER lumen by inhibition of MTP that primarily facilitated the naringenin-mediated decrease in apoB secretion [43, 44]. In detailed pulse-chase studies, naringenin decreased apoB100 secretion from HepG2 cells even in the presence of oleate, through enhanced apoB degradation [44]. Studies in rat liver McARH7777 cells, transfected with constructs containing human apoB of various lengths, revealed that naringenin only inhibited the secretion of apoB-containing particles that required lipidation, whereas shorter length apoB constructs, which require minimal lipidation for secretion, were unaffected [43]. These* in vitro *findings suggest that naringenin limits TG availability for apoB secretion.

Metabolic studies in *Ldlr^−/−^* mice demonstrated that a western type diet stimulated the production rate of VLDL-TG and apoB100 when compared with chow-fed mice [45]. Supplementation of the western diet with 3% naringenin completely prevented the overproduction of apoB100 and TG, supporting the previous *in vitro* data that naringenin prevents VLDL-apoB100 secretion. The pattern of hepatic lipid accumulation mirrored TG secretion, as hepatic TG was increased 2-fold in western-fed animals compared to chow-fed mice and levels were significantly reduced by naringenin. Significant increases in the hepatic mRNA expression of *Srebf1c* (5-fold) and increased TG synthesis (1.3-fold) were related to the significant hyperinsulinemia induced by the western diet. These parameters were all normalized by 3% naringenin. In concert with the decrease in insulin-stimulated lipogenesis, naringenin stimulated hepatic expression of *Cpt1*α*, Acox*, and fatty acid *β*-oxidation to prevent hepatic TG accumulation [45]. In ICR rats fed 1% naringenin for 21 days, significantly increased hepatic levels of mRNA coding for enzymes involved in peroxisomal and omega fatty acid oxidation were observed [46]. Although a pharmacological dose was used in these studies, concentrations of dietary naringenin as low as 0.6% have been reported to lower hepatic triglyceride levels in rats fed a high-fat diet; however, these effects were not observed in rats fed a high carbohydrate diet [47].

In high-fat fed *Ldlr^−/−^* mice, addition of nobiletin (0.1% and 0.3% w/w) resulted in a dramatic reduction in both hepatic and intestinal TG accumulation, attenuation of VLDL-TG secretion, normalization of insulin sensitivity and conferred an almost complete resistance to obesity, without effect on caloric consumption or fat absorption [48]. These studies indicate that prevention of the hepatic lipid load by nobiletin limits the availability lipid for hepatic storage, lipoprotein secretion and deposition in peripheral tissues. Others have demonstrated in hamsters fed a semipurified diet that supplementation with mixtures of 1% polymethoxylated flavonoids, containing tangeretin and nobiletin, lowered plasma concentrations of both TG and cholesterol and reduced hepatic TG [49].

Similar to naringenin, addition of nobiletin to a western diet increased hepatic *β*-oxidation, contributing to reduced hepatic TG, decreased VLDL secretion and the correction of insulin resistance. Both naringenin and nobiletin increased hepatic *Pgc1*α**mRNA, which coincided with increased *Cpt1*α**mRNA, mitochondrial DNA and hepatic FA oxidation [45]. However, nobiletin did not increase mRNA of *Acox* [48]. Studies in male ICR rats fed a diet containing 1% naringenin for 21 days demonstrated increased mRNA levels of enzymes involved in peroxisomal fatty acid oxidation which resulted in a significant increase (+56%) in the rate of peroxisomal fatty acid oxidation compared with controls [50].

The molecular signals for the induction of FA oxidation in liver by naringenin or nobiletin have not been identified. In HepG2 cells, using concentrations of naringenin and nobiletin that inhibited apoB100 secretion by 50–70% (100 *μ*M naringenin, 10 *μ*M nobiletin), we observed upregulation of PPAR*α* target genes including *Cpt1*α**, but not activation of a luciferase promoter containing three PPRE elements suggesting these flavonoids do not function as direct PPAR*α* activators [45, 48]. Furthermore, induction of *Ppar*α**mRNA expression and increased liver weight, characteristic of PPAR*α* activators in mice (fibrates), did not occur with naringenin [45] or nobiletin [48].

In contrast, naringenin has been reported to stimulate PPRE promoter activity in U-2OS cells, a human osteosarcoma cell line [51]. Also, in the human liver cell line Huh-7, naringenin, at relatively high doses, induced PPRE activity 17% at 150 *μ*M and activated a PPAR*α*-GAL4 fusion protein by 24% at 240 *μ*M. These authors also observed increased transcription of enzymes involved in fatty acid utilization including *Pgc1*α**, *Acox*, and *Ucp1* [52]. In a cell free system, naringenin did not increase the binding of PGC1*α* to PPAR*α* but did significantly increase* Pgc1*α** mRNA by 14-fold in the Huh7 hepatocyte cell line [52]. These data suggest that at high concentrations, these flavonoids may directly activate PPAR*α*. However, in human studies oral administration of naringenin in capsules (135–199 mg) or ingestion of grapefruit juice (single dose, 8 mL of juice/kg) leads to much lower plasma naringenin concentrations (6.0–7.3 *μ*mol/L) [53, 54] than concentrations used *in vitro* (50–240 *μ*M), suggesting suboptimal doses for clinical efficacy. Therefore, further experimentation should focus on more closely linking *in vivo* observations with *in vitro* mechanistic studies.

Tangeretin also has been shown to increase hepatic protein concentrations of PPAR*α* which was associated with increased fatty acid oxidation and a reduction in liver triglyceride [55]. Addition of a mixture of polymethoxylated flavonoids (1 : 1; tangeretin : nobiletin) to the diet of fructose-fed Golden Syrian hamsters reduced plasma TG and prevented lipid accumulation in liver and heart, but not in muscle [56]. These authors demonstrated a significant increase in hepatic PPAR*α* protein expression, suggesting that an increase in fatty acid oxidation leads to the observed attenuation of hepatic steatosis and improved dyslipidemia [56].

## 5. Amelioration of Insulin Resistance by Citrus Flavonoids

Skeletal muscle is the major tissue responsible for insulin-mediated glucose uptake. In states where FA flux is increased, as in insulin resistance, both the oxidation of glucose and its conversion to glycogen are impaired [57]. Myocytes and hepatocytes can become overwhelmed by lipid, with storage and oxidation pathways at capacity, allowing FA intermediates such as ceramide and diacylglycerol to accumulate [58, 59]. FA metabolites activate serine kinase pathways including JNK and PLC which can lead to inhibition of the insulin receptor [60], preventing its activation and downstream signaling. Without activation of PI3K, GLUT4 does not undergo translocation and fusion with the plasma membrane resulting in impaired glucose uptake and utilization [61]. *Ppar*α*^−/−^*  mice fed a high-fat diet have increased TG in adipose and muscle, but do not develop insulin resistance, suggesting that PPAR*α*-stimulated oxidation of ectopic lipid in peripheral tissues, including muscle, increases the cellular concentration of lipid derivatives which contribute to insulin resistance [35]. Therefore, treatments that promote glucose utilization by preventing cellular accumulation of TG- and FA-derived lipid metabolites may substantially improve peripheral insulin resistance and ameliorate impaired glucose uptake.

**Figure 2:**
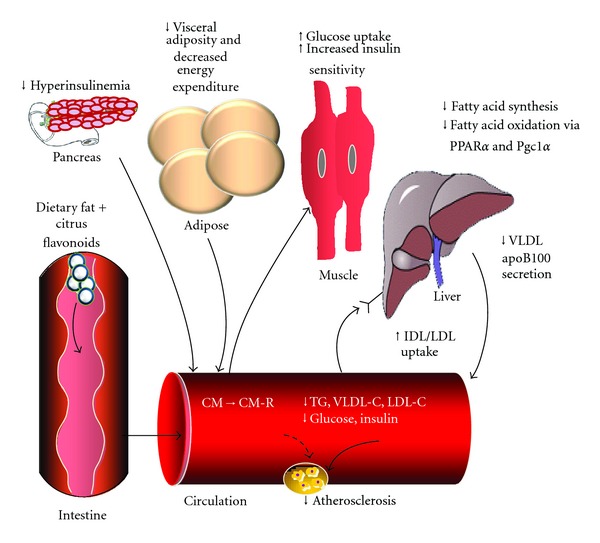
Potential mechanisms for the reduction in risk factors for atherosclerosis by flavonoids. Both *in vitro* and *in vivo *data suggest that citrus flavonoids decrease macrophage uptake of oxidized LDL and macrophage CE accumulation and improve dyslipidemia. While the complete mechanisms have not been fully defined, the nuclear hormone receptors PPAR*α* and PGC1*α* represent important molecular targets. The improvement in dyslipidemia can be linked to decreased VLDL secretion as hepatic lipid availability for storage or VLDL secretion is decreased as a consequence of PPAR-stimulated fatty acid oxidation. The oxidation of fatty acids by liver prevents ectopic lipid accumulation and improves both insulin sensitivity and glucose tolerance. Arrows indicate change in response to flavonoid treatment.

Interestingly, in western diet-fed *Ldlr^−/−^* mice, naringenin did not stimulate fatty acid oxidation in muscle nor were any changes observed in the expression of genes linked to fatty acid oxidation, such as *Cpt1*β**, *Ucp1*, and *Ucp3* [45]. Instead, naringenin inhibited SREBP1c-mediated *de novo* lipogenesis and promoted glucose uptake. These results suggest that naringenin does not act directly on muscle. Reduced muscle lipid accumulation and improved glucose utilization in this model is consequent to attenuated *de novo* lipogenesis and decreased uptake of lipoprotein-derived lipid, the latter a consequence of decreased VLDL secretion. By increasing hepatic FA oxidation, naringenin permits glucose to be utilized by peripheral tissues, including muscle, resulting in the prevention of both lipid accumulation and insulin resistance (Figure 2). The protective effect of naringenin in preventing the metabolic disturbances associated with high-fat feeding was also observed in wild-type C57BL/6J mice. In this model, naringenin significantly reduced plasma and hepatic lipids, normalized glucose tolerance and insulin sensitivity and prevented obesity when compared to western-fed mice, although the time course for the preventative effect was significantly longer than that required for *Ldlr^−/−^* mice [45].

## 6. Citrus Flavonoids as Antioxidants and Free Radical Scavengers

Flavonoids, as dictated by their structure, are reducing agents and can serve as efficient chelators of transition metals involved in cellular oxidation reactions [62, 63]. The association between flavonoids, including citrus flavonoids, and metabolic disorders and atherosclerosis has been linked to their antioxidant properties and to a reduction in oxidative stress. *In vitro*, naringin is an efficient free radical scavenger, but has limited antioxidant activity in mouse liver homogenates [64]. In STZ diabetic rats, naringin (40 mg/kg/day) was shown to prevent diabetic neurogenic pain through its prevention of free radical formation and antioxidant activities in the sciatic nerve [65]. In mice fed a high-fat diet, naringin (0.02%) improved indices of insulin resistance, which was attributed, in part, to improved hepatic enzymes involved in free radical scavenging and antioxidant activities [66]. Naringenin has also been demonstrated to have antioxidant properties. Naringenin (0.02%) increased hepatic glutathione peroxidase and superoxide dismutase activities and decreased hepatic and plasma TBARS [67]. In diabetic rats, naringenin (50 mg/kg/day) significantly increased enzymes with antioxidant activity in pancreas and plasma and decreased serum activities of ALT, AST, ALP, and LDH [68]. However, it is not known if naringenin or naringin has direct effects on the expression of activities of these enzymes, or if the effects are secondary to the flavonoid-induced correction of dysregulated lipid metabolism.

## 7. Prevention of Atherosclerosis by Citrus Flavonoids

In addition to correction of dyslipidemia and glucose intolerance, treatment with nobiletin (0.3% w/w) or naringenin (3% w/w) in *Ldlr^−/−^* mice for 6 months led to a marked reduction in the progression of atherosclerosis [48, 69]. In rabbits fed a high-cholesterol diet, supplementation of 0.1% naringin or 0.05% naringenin for 8 weeks decreased aortic fatty streak area when compared to controls [70]. In wild-type mice fed a high-fat/high-cholesterol/0.5% cholic acid in a cocoa butter diet, 0.02% naringin reduced plaque progression by 41%, but no beneficial effects of naringin were observed in *ApoE^−/−^* mice fed the same diet [71]. Naringin significantly reduced soluble E-selectin and soluble inter-cell adhesion molecule-1 in these mice [71]. Molecular studies have demonstrated that incubation of lipopolysaccharide-treated RAW-264.7 macrophages with naringenin, dose-dependently decreased the production of tumour necrosis factor (TNF*α*) and monocyte chemoattractant protein 1 (MCP-1) [72]. Nobiletin and its derivatives have been shown to reduce the expression and activity of macrophage scavenger receptors [73] including CD36 and SRAI/II and decrease the uptake of acetylated LDL [74]. These studies suggest that in addition to decreased exposure of the vessel wall to increased plasma lipoproteins, flavonoids directly decrease both lipoprotein uptake and the inflammatory response within macrophages of the arterial intima. PPAR*α* activation has also been demonstrated to reduce monocyte recruitment to the vascular wall by downregulating vascular cell adhesion molecule-1, MCP-1 and interleukin-6 [75]. Ligands of PPAR*α* have also been demonstrated to reduce foam cell formation by reducing lipid uptake and increasing lipid efflux [76]. Fenofibrate has been demonstrated to reduce foam cell formation and slow the progression of atherosclerosis in mice [77, 78], suggesting that some of the effects of citrus flavonoids may be mediated through PPAR*α*-stimulated pathways.

## 8. Additional Targets of Citrus Flavonoids

While some preliminary human studies suggest metabolic benefit [39–41], an enhanced understanding of the molecular targets of natural polyphenols is required in order to identify the main mechanism of action. While PPAR*α* has emerged as a potential target, numerous studies have also identified PPAR*γ* as a prospective target. In diabetic male Wistar albino rats fed a high-fat diet, naringin increased PPAR*γ* protein expression in liver. Interestingly, the authors also observed a decrease in the expression of LXR and SREBP1c and a reduction in hepatic steatosis [79]. It has also been reported that naringenin at 80 *μ*M, activated a PPAR*γ*-GAL4 construct up to 57% [52]. In 3T3-L1 adipocytes, both naringenin and hesperitin upregulated PPAR*γ* at both 80 *μ*M and 160 *μ*M, which resulted in a significant increase in the expression of adiponectin [51]. Nobiletin (10 *μ*M) stimulates differentiation of ST-13 preadipocytes into mature adipocytes and significantly increases the production of adiponectin protein [80].

## 9. Summary

Collectively, these studies add to the growing body of evidence that citrus flavonoids have marked lipid and lipoprotein lowering potential and demonstrate that naringenin and nobiletin reduce hepatic lipid accumulation and prevent lipoprotein overproduction, normalize glucose tolerance and insulin sensitivity and slow the progression of atherosclerosis (Figure 2). These beneficial metabolic effects are mediated, in part, by the metabolic regulators, PPAR*α*, PPAR*γ*, and PGC1*α*, although further studies are required to fully reveal the interaction of these flavonoids with upstream mediators in this pathway.

## Abbreviations

ACOX: Acyl-CoA oxidase
ACAT: Acyl-CoA:cholesterol acyltransferase
apoB100: Apolipoprotein B100
CPT1: Carnitine palmitoyl transferase 1
CE: Cholesteryl ester
CVD: Cardiovascular disease
DGAT1/2: Diacylglycerol acyl transferase 1/2
FA: Fatty acid
FAS: Fatty acid synthase
FC: Free cholesterol
HDL: High density lipoprotein
LDL: Low density lipoprotein
MCP: Monocyte chemmoattractant protein 1
MTP: Microsomal triglyceride transfer protein
PI3K: Phosphoinositide-3-kinase
PPAR: Peroxisome proliferator-activated receptor
PPRE: PPAR response element
PGC1*α*: Peroxisome proliferator-activated receptor gamma coactivator 1*α*
SREBP1c: Sterol regulatory element binding protein
TG: Triglyceride
TNF*α*: Tumour necrosis factor
VLDL: Very low density lipoprotein
UCP-1: Uncoupling protein 1.
